# Single Nucleotide Polymorphism in Gene Encoding Transcription Factor Prep1 Is Associated with HIV-1-Associated Dementia

**DOI:** 10.1371/journal.pone.0030990

**Published:** 2012-02-07

**Authors:** Sebastiaan M. Bol, Thijs Booiman, Daniëlle van Manen, Evelien M. Bunnik, Ard I. van Sighem, Margit Sieberer, Brigitte Boeser-Nunnink, Frank de Wolf, Hanneke Schuitemaker, Peter Portegies, Neeltje A. Kootstra, Angélique B. van 't Wout

**Affiliations:** 1 Department of Experimental Immunology, Sanquin Research, Landsteiner Laboratory and Center for Infection and Immunity Amsterdam (CINIMA) at the Academic Medical Center of the University of Amsterdam, Amsterdam, The Netherlands; 2 HIV Monitoring Foundation, Academic Medical Center of the University of Amsterdam, Amsterdam, The Netherlands; 3 Department of Infectious Disease Epidemiology, Imperial College, London, United Kingdom; 4 Department of Neurology at the Academic Medical Center of the University of Amsterdam, Amsterdam, The Netherlands; 5 Department of Neurology at the OLVG Hospital, Amsterdam, The Netherlands; Institut National de la Santé et de la Recherche Médicale, France

## Abstract

**Background:**

Infection with HIV-1 may result in severe cognitive and motor impairment, referred to as HIV-1-associated dementia (HAD). While its prevalence has dropped significantly in the era of combination antiretroviral therapy, milder neurocognitive disorders persist with a high prevalence. To identify additional therapeutic targets for treating HIV-associated neurocognitive disorders, several candidate gene polymorphisms have been evaluated, but few have been replicated across multiple studies.

**Methods:**

We here tested 7 candidate gene polymorphisms for association with HAD in a case-control study consisting of 86 HAD cases and 246 non-HAD AIDS patients as controls. Since infected monocytes and macrophages are thought to play an important role in the infection of the brain, 5 recently identified single nucleotide polymorphisms (SNPs) affecting HIV-1 replication in macrophages *in vitro* were also tested.

**Results:**

The *CCR5* wt/Δ32 genotype was only associated with HAD in individuals who developed AIDS prior to 1991, in agreement with the observed fading effect of this genotype on viral load set point. A significant difference in genotype distribution among all cases and controls irrespective of year of AIDS diagnosis was found only for a SNP in candidate gene *PREP1* (*p* = 1.2×10^−5^). Prep1 has recently been identified as a transcription factor preferentially binding the −2,518 G allele in the promoter of the gene encoding MCP-1, a protein with a well established role in the etiology of HAD.

**Conclusion:**

These results support previous findings suggesting an important role for MCP-1 in the onset of HIV-1-associated neurocognitive disorders.

## Introduction

While the prevalence of HIV-1-associated dementia (HAD) has greatly decreased, first with the introduction of zidovudine [1], [2] and later with combination antiretroviral therapy (cART) [3], [4], neurocognitive impairment is still seen more frequently in HIV-1-infected patients than in seronegative individuals. In recent years a new terminology has been developed to classify this broadening clinical spectrum of neurocognitive impairment, including milder abnormalities. HAND (HIV-1-associated neurocognitive disorders) is the umbrella definition, comprising three entities: asymptomatic neurocognitive impairment, mild neurocognitive disorders (MND), and HAD. Clinical symptoms of HAND are cognitive impairment (memory, concentration), motor dysfunction and behavioral changes. Recent studies showed that MND occurred in 15–50% of the HIV-1-infected individuals [5]–[9], and HAD in 1–10% of the patients [4], [5], [7], [8].

Although CD4+ T cells are the predominant cell type infected by HIV-1 and primarily associated with the disease course, circulating monocytes as well as macrophages can also become infected and contribute to the viral reservoir and disease progression [10]. Furthermore, monocytes and macrophages play a crucial role in certain HIV-1-related pathologies, including HAND [10]. Despite lack of strong evidence it is generally believed that HIV-1 migrates across the blood-brain barrier in monocytes that were infected in the blood [11], [12]. Indeed, in the brain, the monocyte-derived perivascular macrophages and microglia are the most commonly HIV-1-infected cells [13], [14]. Complex mechanisms underlie the neurodegeneration, since neurons themselves are not infected by HIV-1. Local production of HIV-1 proteins [15]–[18] or other non-HIV compounds [19]–[24] by infected and activated macrophages and microglia cause neuronal damage. Furthermore, neuronal injury may occur as a consequence of the inflammatory process in the brain [25]–[27].

As is the case for many complex disorders, it remains unclear why some individuals are more at risk to develop HAND than others. The cause of the neurodegeneration is multi-factorial, and in addition to viral genetic factors [28]–[30], host genetic predisposition may also contribute to the susceptibility to these disorders. We previously reported a reduced prevalence of the 32 base pair deletion in the *CCR5* gene in HIV-infected individuals with HAD as compared to controls with AIDS but no HAD [31], and recently a single nucleotide polymorphism (SNP) in the gene of one of its natural ligands, *CCL3*, was identified to be associated with HAD as well [32]. SNPs in *MCP-1* (monocyte chemoattractant protein-1) and *TNFA* were found to affect protein expression levels of these genes [33]–[36] and were associated with the onset of HAD [34], [37], [38]. A biological mechanism by which the SNP in *MCP-1*, located at position −2,518 in the promoter region, affects gene expression was described recently [39]. This study demonstrated that the transcription factor Prep1 preferentially binds the −2,518 G allele in *MCP-1*, thereby affecting transcription of this protein. Furthermore a SNP in *CCR2*, encoding the receptor for MCP-1, was associated with rate of progression to neuropsychological impairment [40]. However, few of the associations between polymorphisms and HAD have been replicated in other studies (see **Table 1** for an overview of all identified associations between host common genetic variants and HAD).

**Table 1:** Overview of all common genetic variants tested for association with HIV-1-associated neurocognitive disorders.

Gene	Polymorphism	Association with HAND	No association with HAND
*APOE*	E4 isoform	Corder *et al.* [60], HAD; prospective cohort study (n = 44)	Pemberton *et al.* [61], HAD; 56 cases, 112 controls
			Diaz-Arrastia *et al.* [62], HIVE; cohort A: 43 cases and 104 controls, and cohort B: 14 cases and 117 controls
			Dunlop *et al.* [63], HAD and HIVE; 32 HAD cases, 24 possible HAD cases and 73 controls
*CCL3*	rs1130371	Levine *et al.* [32], HAD; 26 cases, 117 controls	
*CCR2*	rs1799864V64I	Singh *et al.* [40], HAND, prospective cohort study (n = 121)	Van Rij *et al.* [31], HAD; 49 cases, 186 controls
*CCR5*	Δ32	Van Rij *et al.* [31], HAD; 49 cases, 186 controls	Singh *et al.* [40], HAND, prospective cohort study (n = 121)
*MCP-1*	rs1024611(−2518 A>G)	Gonzalez *et al.* [34], HAD; prospective cohort study (n = 1,115)	Singh *et al.* [40], HAND, prospective cohort study (n = 121)
		Shiramizu *et al.* [37], HIV-1 DNA in CSF; 27 specimens	Levine *et al.* [32], HAD; 26 cases, 117 controls
*TNFA*	rs1800629(−308 G>A)	Quasney *et al.* [38], HAD; 16 cases, 45 controls	Sato-Matsumura *et al.* [64], HIVE; 44 cases, 30 controls
		Pemberton *et al.* [61], HAD; 56 cases, 112 controls	Levine *et al.* [32], HAD; 26 cases, 117 controls
			Diaz-Arrastia *et al.* [62], HIVE; cohort A: 43 cases and 104 controls, and cohort B: 14 cases and 117 controls

HAND, HIV-1-associated neurocognitive disorders; HAD, HIV-1-associated dementia; HIVE, HIV-1 encephalitis.

Here, we evaluated the polymorphisms in previously tested candidate genes *CCR5*, *CCR2*, *MCP-1*, *TNFA*, *APOE* and *CCL3*, as well as a polymorphism in the novel candidate gene *PREP1*, for their association with HAD in participants of the Amsterdam Cohort Studies of HIV infection and AIDS (ACS) and the AIDS therapy evaluation in The Netherlands (ATHENA) observational cohort. In addition, given the important role of monocytes and macrophages in the etiology of HAD, SNPs that we recently identified to affect HIV-1 replication in macrophages *in vitro* were also tested [41].

## Materials and Methods

### Ethics statement

This study has been conducted in accordance with the ethical principles set out in the declaration of Helsinki. Anonymized archival material (peripheral blood mononuclear cells or DNA) from AIDS patients was used in this study, which was approved by the Medical Ethics Committee of the Academic Medical Center in Amsterdam, The Netherlands. For the qPCR experiments, anonymized buffy coat or full blood was used from healthy blood donors, and the use was approved by the Medical Ethics Committee of the Academic Medical Center and the Ethics Advisory Body of the Sanquin Blood Supply Foundation in Amsterdam, The Netherlands. Written informed consent was obtained from all of these healthy donors.

### Study population

In total we selected 86 AIDS patients with HAD (cases) from the Amsterdam Cohort Studies and ATHENA observational cohort from whom DNA was available for genotyping. Before the AIDS dementia complex was defined as a distinct clinical syndrome the diagnosis of dementia in these patients had been based on DSM-III (Diagnostic and Statistical Manual of Mental Disorders) criteria. Motor abnormalities were present in all these demented patients, which agreed retrospectively with the diagnostic criteria of the AIDS dementia complex [42]. When more precise diagnostic criteria were introduced in 1991 and 2007 ([43], [44]), these criteria were used to classify these patients. The absence of dementia was confirmed by neurological examination by a neurologist.

We compared the HAD patients with 246 AIDS patients without HAD (controls) (**Table 2**). A subset of these samples (49 cases and 186 controls) was included in a previous study that investigated *CCR5* Δ32 and *CCR2* V64I genotype frequencies between HAD cases and controls [31].

**Table 2:** Characteristics of the studied population consisting of AIDS patients with or without HAD.

Characteristics	HAD patients	Non-HAD patients	*p*
	(cases, n = 72)	(controls, n = 241)	
AIDS diagnosis (year); median (range)	1989 (1984–2005) n = 69	1990 (1985–2005) n = 241	–
Time AIDS to death or start cART (months); median (range)	14 (0–114) n = 67	12 (0–81) n = 234	0.21^1^
Time AIDS to HAD (months); median (range)	5 (0–114) n = 69	N.A.	<0.0001^1^^,^ 2
Age at diagnosis AIDS; average (range)	40 (22–63) n = 69	41 (23–71) n = 241	0.60^3^
CD4+ T cell count (cells/µl) at AIDS, median (range)^4^	120 (10–850) n = 39	105 (7–1,380) n = 166	0.48^1^
Mode of HIV-1 transmission (IDU : other)	4 : 35	4 : 158	0.048^5^

N.A., not applicable; HAD, HIV-1-associated dementia; IDU, injecting drug user.

1 Mann Whitney test.

2 Time to develop HAD after AIDS diagnosis among the cases was compared to the time from AIDS diagnosis to death or to start cART in the control group.

3 unpaired t test.

4 CD4+ T cell counts within 6 months to the date of AIDS diagnosis.

5 Fisher's exact test.

**Figure 1:**
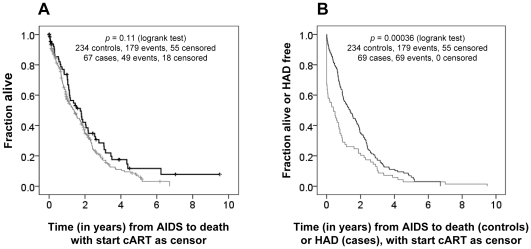
Comparison of AIDS survival between HAD cases and non-HAD controls. (**A**) Kaplan Meier analysis for time from AIDS to death with start cART as censor (vertical lines), for both HAD cases (grey line) and non-HAD AIDS patients as controls (black line). (**B**) Kaplan Meier analysis for time from AIDS to death with start cART as censor (vertical lines) for the controls (black line) and for time from AIDS to HAD for the HAD cases (grey line). cART, combination antiretroviral therapy; HAD, HIV-1-associated dementia.

Cases and controls were matched for year of AIDS diagnosis (AIDS diagnosis was based on AIDS defining events according to the CDC AIDS definition 1987; Kaposi's sarcoma was excluded as an AIDS defining event), time from AIDS diagnosis to death or to start of combination antiretroviral therapy (cART), age at AIDS diagnosis and CD4+ T cell count at AIDS diagnosis. Cases receiving cART more than 6 months before their HAD diagnosis (n = 14), as well as controls who started cART more than 6 months before their AIDS diagnosis (n = 5) were excluded from the analysis. Median time from AIDS to developing HAD for the cases was significantly shorter than the time from AIDS to death or to start cART in the control population (*p*<0.0001; Mann Whitney test) (**Table 2**), indicating that time from AIDS to death or to start cART for the HIV-1-infected individuals in the control group was in principle long enough to develop HAD. Similar results were obtained when using Kaplan-Meier analysis, with no difference in time from AIDS to death (start cART used as censor) between cases and controls (*p* = 0.11, logrank test) and significant shorter time from AIDS to HAD for the cases, than time from AIDS to death (start cART as censor) for the controls (*p* = 0.00036, logrank test) (**Figure 1A–B**). Information on ancestry was only known for a limited number of patients and was based on reported ethnicity by the treating physician or reported country of birth.

### Candidate SNP selection and genotyping

Genotype distributions of polymorphisms previously associated with HAND (**Table 1**) as well as SNPs associated with *in vitro* HIV-1 replication in macrophages (cutoff *p* value = 5×10^−5^) (**Table S1**) [41], were analyzed in this case-control study. *PREP1* was selected because of its preferred binding to the −2,518 G allele in the promoter region of *MCP-1*. SNP rs2839619 was selected from 17 SNPs in *PREP1* after preliminary analysis comparing 15 cases with 126 unmatched controls. This SNP seems of particular interest since it was shown to be associated with cholesterol metabolism [45], which is known to play a role in the etiology of Alzheimer's disease [46] and in addition, is in linkage disequilibrium (r^2^ = 0.51) with nearby intronic SNP rs234720 that has been associated with cognitive test performance [47].

Peripheral blood mononuclear cells were used for isolation of genomic DNA using QIAamp DNA blood mini kit (Qiagen, Valencia, CA, USA) or NucleoSpin blood kit (Macherey-Nagel, Dueren, Germany). SNP genotypes for SNPs in *DYRK1A*, *PDE8A*, *UBR7* and *PREP1* (**Table S1**) were available for 172 individuals (18 cases, 154 controls) from a recent study on the effects of host genetic variation on HIV-1 susceptibility and disease progression [48]. For the remaining DNA samples and other SNPs not present on the Illumina SNP beadchip, ABI TaqMan® SNP genotyping assays (Applied Biosystems, Carlsbad, CA, USA) were used for genotyping (**Table S1**). For all SNPs except the SNP in *TNFA*, genotyping assays were run on a LightCycler® 480 system (Roche, Basel, Switzerland) using Probes Master (Roche) with the following amplification cycles: 10 min 95°C; 50 cycles of 15 sec 95°C, 1 min 60°C.

The *TNFA* SNP assay was run on an Applied Biosystems 7500 Fast Real-Time PCR System (Applied Biosystems) with Taqman genotyping master mix (Applied Biosystems), and using the following amplification cycles: 10 min 95°C; 40 cycles of 15 sec 95°C, 1 min 60°C. *APOE* allele types (E2, E3 or E4 [49]) were determined by genotyping SNPs rs429358 (C or T) and rs7412 (C or T). *CCR5* Δ32 and *CCR2* V64I genotyping was performed as described previously [50], [51].

### Quantitative PCR

Buffy coat or full blood was obtained from 69 healthy blood donors. Monocyte isolation and monocyte-derived macrophage (MDM) culture was performed as previously described [52]. Total RNA was extracted from day 7 uninfected MDM using the High Pure RNA Isolation kit (Roche). Oligo(dT) primers were used for reverse transcription of mRNA, using Roche's Transcriptor First Strand cDNA Synthesis kit (60 min at 50°C). Resulting cDNA was used for quantitative PCR (qPCR) analysis, using the following primes: PREP1 F and R, MCP-1 F and R, and GAPDH F and R (**Table S2**). qPCRs were performed with SYBR Green I Master (Roche) and were run on a LightCycler® 480 system (Roche) using the following amplification cycles: 10 min 95°C; 50 cycles of 10 sec 95°C, 20 sec 58°C, 30 sec 72°C. All procedures were carried out according to manufacturer's protocol. Messenger RNA expression is reported relative to *GAPDH*. Gene expression values were obtained using Roche's LightCycler® relative quantification software (release 1.5.0). To facilitate accurate and reliable between-donor comparison, cDNA synthesis and qPCR experiments for all 69 samples were performed simultaneously.

### Statistical analysis

Fisher's exact test was used to test for differences in SNP genotype distribution between cases and controls. To test for differences in group characteristics between cases and controls the Mann Whitney test and the unpaired t test were used. One-way ANOVA was performed to test for differences in mRNA levels between genotypes. Statistical analysis was performed using the statistical computing software R (version 2.9.0) and GraphPad Prism (version 5).

## Results

Genotype frequencies for all polymorphisms tested, in the group of cases with HAD (n = 72) and the group of controls that did not develop HAD (n = 241), are displayed in **Table 3**. Of the 12 polymorphisms tested, a significant difference in genotype distribution for SNP rs2839619 in *PREP1* was found between cases and controls (*p* = 1.2×10^−5^; 71 cases and 235 controls; DNA was limited for some of the cases or controls, therefore not all samples could always be genotyped). The difference remained statistically significant after Bonferroni correction for multiple comparisons (n = 12), p = 1.4×10−4. Also after excluding HAD cases with known non-Caucasian ethnicity, or for whom injecting drug use was reported as mode of HIV-1 transmission, a known risk factor for HAD [4], the difference in *PREP1* SNP genotype distribution between cases (n = 64) and controls remained significant (n = 211) (*p* = 4.3×10^−5^). Quantitative PCR experiments performed to investigate if the SNP in *PREP1* was associated with either *PREP1* or *MCP-1* mRNA levels showed no difference in *PREP1* or *MCP-1* mRNA levels between the three *PREP1* SNP genotypes (*p* = 0.3 and 0.8, respectively, n = 69; one-way ANOVA) (data not shown).

**Table 3:** Genotype distribution among HAD (cases) and non-HAD (controls) HIV-1-infected patients for all polymorphisms tested.

Gene	Polymorphism	Cases (HAD)			Controls (no HAD)			*p*
		AA	AB	BB	AA	AB	BB	
*APOE* 1	E4 isoform^2^	52	16	1	158	52	5	0.95
*CCL3* 1	rs1130371	40	26	5	133	90	9	0.53
*CCR2* 1	rs1799864 (V64I)	64	8	0	206	34	1	0.66
*CCR5* 1	Δ32	66	6	0	203	38	0	0.13
*DYRK1A* 3	rs12483205	38	25	8	125	96	11	0.13
*MCP-1* 1	rs1024611 (−2518 A>G)	3	27	41	14	101	116	0.58
*MOAP1* 3	rs1046099	29	36	6	117	93	20	0.28
*PDE8A* 3	rs12909130	33	34	4	110	96	26	0.34
***PREP1***	**rs2839619**	30	17	24	55	130	50	**1.2×10^−5^** *
*SPOCK3* 1	rs17519417	21	32	17	76	102	53	0.91
*TNFA* 1	rs1800629 (−308 G>A)(−308 G>A)	57	13	1	158	62	9	0.20
*UBR7* 3	rs2905	10	28	33	22	107	103	0.42

1 Polymorphisms selected from earlier studies that tested for association between genotype and HAD.

2 In the case of *APOE* AA, AB and BB refer to no APO E4, one APO E4 allele and two APO E4 alleles, respectively.

3 SNPs selected from a previous study that found associations between these SNPs and HIV-1 replication in macrophages.

*Significant difference after correction for multiple testing (n = 12); Bonferroni threshold *p* = 4.2×10^−3^.

Remarkably, for none of the other polymorphisms tested, including those previously reported to be associated with HAD (*CCR5* Δ32, promoter SNP in *MCP-1*, the −308 G>A SNP in *TNFA*, *CCR2* V64I variant, Apo E4 isoform and a SNP in *CCL3*), a significant difference was found in genotype distribution between cases and controls. In a previous case-control study [31], we described a reduced prevalence of the *CCR5* wt/Δ32 genotype among HAD patients. A subset of cases and controls from this published study overlaps with our current study population. Cases and controls that were additionally included for this study seroconverted on average later in time. We recently reported that in the HIV-1 epidemic in The Netherlands, the impact of certain host factor polymorphisms, including *CCR5* Δ32, might be fading [53]. We therefore hypothesized that the protective impact of the *CCR5* wt/Δ32 genotype on the onset of HAD also may have decreased over time. To test this hypothesis we divided cases and controls into two groups using the median year of AIDS diagnosis of the complete study population (1990). This approach was chosen over using seroconversion date since this information was unavailable for 29 of 42 cases with AIDS diagnosis ≤1990. Importantly, cases and controls with AIDS diagnosis ≤1990 as well as with AIDS diagnosis >1990 matched all of the characteristics as described above (**Table S4**), and time from AIDS to death or to start cART for the HIV-1-infected individuals in the control group was in principle long enough to develop HAD (*p* = 0.005 and *p* = 0.001 for cases and controls with AIDS diagnosis ≤1990 and >1990, respectively, Mann Whitney test) (**Table S4**). Similar results were obtained when using Kaplan-Meier analysis and logrank test (data not shown). When the *CCR5* wt/Δ32 genotype frequency was compared between cases and controls we observed a difference in the “AIDS diagnosis ≤1990” group (*p* = 0.046), but not for the “AIDS diagnosis >1990” group (*p* = 1.00) (**Table S3**), indeed suggestive of a fading protective effect. Assuming that other genetic effects may also have diminished over time in our cohort, we performed the same analysis for the remaining polymorphisms (**Table S3**). For 9 polymorphisms, no significant associations were observed in either group. The effect of the SNP in *PREP1* was clearly independent of the year of AIDS diagnosis, since in both groups there were significantly fewer heterozygous individuals for SNP rs2839619 among cases than controls (*p* = 0.001 and *p* = 0.008 for the “AIDS diagnosis ≤1990” and the “AIDS diagnosis >1990 group”, respectively) (**Table S3**). Conversely, a significant (defined as *p*<0.05; however not significant after correction for multiple testing, n = 24) association with HAD was observed for the SNP in *DYRK1A* only in the group with AIDS diagnosis after 1990.

## Discussion

Here we describe the first combined evaluation of all previously identified genetic polymorphisms reported to be associated with the prevalence of HAND. In addition, we evaluated polymorphisms that we recently identified to be associated with HIV-1 replication in macrophages for their association with HAD. For one of the 12 polymorphisms tested, SNP rs2839619 in *PREP1*, we observed a significantly different genotype distribution when comparing AIDS patients with and without HAD. The prevalence of the heterozygous genotype was 55% among controls (and 53% in the HapMap CEU population, n = 226), as compared to only 24% among HAD cases, suggesting that the heterozygous genotype has a protective effect against the development of HAD (positive heterosis). Although multiple examples of heterosis exist ([54], [55] and reviewed in [56]), the molecular basis for this heterozygous effect sometimes remains difficult to understand (reviewed in [56], [57]).

Case-control studies are greatly influenced by variation in allele frequency across different subgroups [58] that may lead to identification of false positive associations. However, the association for the *PREP1* SNP remained significant after excluding patients expected to be of non-European descent. Moreover, since the allele frequency for this SNP is similar for Caucasians, Asians and Africans (NCBI dbSNP) we do not expect that additional population stratification resulting from ethnicity would affect this association. Differences in population substructure may be of importance when SNP rs2839619 is not the causal variant but rather tags another genetic variant, since for that particular SNP genotype distributions may vary between different populations. In addition, the outcome remained unaffected after correcting for injecting drug use as mode of HIV-1 transmission. Although it is known that Prep1 binds to the promoter region of *MCP-1*, we were unable to demonstrate an association between SNP genotype and *MCP-1* mRNA levels in MDM. However, MCP-1 is secreted by monocytes and macrophages, but expression is not limited to these cell-types. The cytokine is also expressed in for HAD possibly more relevant cells such as endothelial cells, astrocytes, microglia and neurons ([59] and references therein). Functional follow-up studies will need to delineate a mechanism that helps to explain the observed reduced frequency of heterozygous donors in the group of HAD patients.

None of the previously identified associations between genetic variants and HAND could be replicated in our present study, even when tested under a dominant or recessive model (data not shown). Many of the candidate gene polymorphisms suggested to play a role in the prevalence of HAND have not been reproduced widely in other cohorts (**Table 1**). Limitations in the availability of patient material, heterogeneity in HAND diagnoses, differences in case-control matching strategies and possible population substructure may have contributed to the absence of robust and replicable results. While we tried to carefully address many of these issues in this study, no robust replication of the reported associations was obtained, suggesting that meta-analyses of multiple HAND cohorts may be required to reliably evaluate the effect of host polymorphisms on HAND.

SNPs previously associated with HIV-1 Gag p24 levels in macrophage cultures were not found to be associated with HAD, although meta-analyses may be required to firmly establish this. This possibly suggests that the quantity of HIV-1 replication in macrophages is less important for the etiology of this phenotype as compared to the immune activation in the brain as a consequence of HIV-1 replication [12].

The protective effect observed for the *CCR5* wt/Δ32 genotype was only observed in the group of individuals that had an AIDS diagnosis ≤1990 and no longer in the group that was diagnosed with AIDS >1990. Excluding cases and control from non-European descent, in whom the *CCR5* wt/Δ32 genotype is less frequent, did not change the outcome of the analysis. In agreement with these findings, we observed a similar fading impact of the *CCR5* wt/Δ32 genotype on HIV-1 control over calendar time and at a population level [53].

We also observed a difference in *DYRK1A* SNP rs12483205 genotype distribution between cases and controls with AIDS diagnosis ≤1990. While the minor allele of this SNP was associated with reduced replication of HIV-1 in monocyte-derived macrophages *in vitro* [41] the observed difference here is the result of 35% more cases homozygous for the major allele, yet 59% fewer cases with the heterozygous genotype as compared to the controls (data not shown). This difference could possibly be due to population stratification since in both Asian and sub-Saharan African populations the frequency of the homozygous major and heterozygous genotype is higher and lower respectively (dbSNP).

The association of a SNP in *PREP1* with the onset of HAD further supports the biological importance of MCP-1 in the pathogenesis of this disease. Replication of this association in an independent cohort using matched HAD cases and controls will now be highly desirable. Functional studies will be required to delineate how the observed difference in allele frequencies can be explained in a biological context.

## Supporting Information

Table S1 **Overview of SNPs genotyped using the ABI TaqMan® SNP genotyping.**
(DOC)

Table S2 **Overview of primers used for qPCR experiments.**
(DOC)

Table S3 **Overview of genotype distribution comparisons between HAD cases and controls with AIDS diagnosis before or after 1991.**
(DOC)

Table S4 **Characteristics of HIV-1-positive patients with or without HAD, divided in a groups with AIDS diagnosis before or after 1991.**
(DOC)
