# Supplementary material for: Single Nucleotide Polymorphism in Gene Encoding Transcription Factor Prep1 Is Associated with HIV-1-Associated Dementia
Source: PLoS One. 2012 Feb 7;7(2):e30990. doi: 10.1371/journal.pone.0030990 (PMC3274517; doi:10.1371/journal.pone.0030990)
Supplement: Table S1 — Overview of SNPs genotyped using the ABI TaqMan® SNP genotyping. (DOC) [file pone.0030990.s001.doc]

**Table S1**. Overview of SNPs genotyped using the ABI TaqMan® SNP genotyping.

| **SNP (gene)** | **ABI assay ID** |
| --- | --- |
| rs429358 (*APOE*) | C__3084793_20 |
| rs7412 (*APOE*) | C__904973_10 |
| rs1130371 (*CCL3*) | C__9458941_10 |
| rs12483205 (*DYRK1A*) ^1^ | C__31609775_10 |
| rs1024611 (*MCP-1*) | C__2590362_10 |
| rs1046099 (*MOAP1*) | C__7585751_10 |
| rs12909130 (*PDE8A*) ^1^ | C__1342209_10 |
| rs2839619 (*PREP1*) ^1^ | C__1605356_1 |
| rs17519417 (*SPOCK3*) | C__33238869_10 |
| rs1800629 (*TNFA*) | C__7514879_10 |
| rs2905 (*UBR7*) ^1^ | C__3236245_20 |

^1^ These SNPs were determined in part of the DNA samples using Illumina SNP beadchips
