# Supplementary material for: Single Nucleotide Polymorphism in Gene Encoding Transcription Factor Prep1 Is Associated with HIV-1-Associated Dementia
Source: PLoS One. 2012 Feb 7;7(2):e30990. doi: 10.1371/journal.pone.0030990 (PMC3274517; doi:10.1371/journal.pone.0030990)
Supplement: Table S2 — Overview of primers used for qPCR experiments. (DOC) [file pone.0030990.s002.doc]

**Table S2**. Overview of primers used for qPCR experiments.

| **Primer name** | **Sequence (5’ – 3’)** |
| --- | --- |
| PREP1 F | GAGTGGCAGGGCTGGCTGTG |
| PREP1 R | GGGGCAGAACCCCCTCTGGA |
| MCP-1 F | TCGCACTCTCGCCTCCAGCA |
| MCP-1 R | TCGCGAGCCTCTGCACTGAG |
| GAPDH F | GTGAAGGTCGGAGTCAACGGGTG |
| GAPDH R | CCGCAGAGCGCGAAAGGAAAGA |
