# Supplementary material for: Single Nucleotide Polymorphism in Gene Encoding Transcription Factor Prep1 Is Associated with HIV-1-Associated Dementia
Source: PLoS One. 2012 Feb 7;7(2):e30990. doi: 10.1371/journal.pone.0030990 (PMC3274517; doi:10.1371/journal.pone.0030990)
Supplement: Table S3 — Overview of genotype distribution comparisons between HAD cases and controls with AIDS diagnosis before or after 1991. (DOC) [file pone.0030990.s003.doc]

**Table S3.** Overview of genotype distribution comparisons between HAD cases and controls with AIDS diagnosis before or after 1991.

| **Gene** | **Polymorphism** | **AIDS diagnosis ≤ 1990** | **AIDS diagnosis > 1990** |
| --- | --- | --- | --- |
|  |  | ***p*** ^1^ | ***p*** ^1^ |
| *APOE* | E4 isoform | 0.86 | 0.86 |
| *CCL3* | rs1130371 | 0.34 | 1.00 |
| *CCR2* | rs1799864 (V64I) | 0.16 | 0.08 |
| *CCR5* | ∆32 | **0.046** | 1.00 |
| *DYRK1A* | rs12483205 | 0.26 | **0.009** |
| *MCP-1* | rs1024611 (-2518 A>G) | 0.96 | 0.11 |
| *MOAP1* | rs1046099 | 0.81 | 0.18 |
| *PDE8A* | rs12909130 | 1.00 | 0.20 |
| ***PREP1*** | **rs2839619** | **0.001** | **0.008** |
| *SPOCK3* | rs17519417 | 0.45 | 0.33 |
| *TNFA* | rs1800629 (-308 G>A) | 0.43 | 0.25 |
| *UBR7* | rs2905 | 0.89 | 0.25 |

HAD, HIV-1-associated dementia

^1^ Fisher’s exact test
