# Supplementary material for: Single Nucleotide Polymorphism in Gene Encoding Transcription Factor Prep1 Is Associated with HIV-1-Associated Dementia
Source: PLoS One. 2012 Feb 7;7(2):e30990. doi: 10.1371/journal.pone.0030990 (PMC3274517; doi:10.1371/journal.pone.0030990)
Supplement: Table S4 — Characteristics of HIV-1-positive patients with or without HAD, divided in a groups with AIDS diagnosis before or after 1991. (DOC) [file pone.0030990.s004.doc]

**Table S4. Characteristics of HIV-1-positive patients with or without HAD, divided in a groups with AIDS diagnosis before or after 1991.**

| **Characteristics** | **AIDS diagnosis ≤ 1990** | | ***p*** | **AIDS diagnosis > 1990** | | ***p*** |
| --- | --- | --- | --- | --- | --- | --- |
|  | Cases (n=42) | Controls (n=125) |  | Cases (n=27) | Controls (n=116) |  |
| AIDS diagnosis (year); median (range) | 1987 (1984–1990) | 1988 (1985–1990) |  | 1995 (1991–2005) | 1994 (1991–2005) |  |
| Time AIDS to death or start cART (months); median (range) | 14 (0­–75) n=41 | 11 (0–81) n=118 | 0.19 ^1^ | 14 (0–114) n=26 | 12 (0–74) n=116 | 0.89 ^1^ |
| Time AIDS to HAD (months); median (range) | 6 (0–51) n=42 | N.A. | 0.005 ^1,2^ | 0 (0–114) n=27 | N.A. | 0.001 ^1,2^ |
| Age at diagnosis AIDS; average (range) | 40 (23–63) n=42 | 39 (23–59) n=122 | 0.88 ^3^ | 41 (22–55) n=27 | 42 (23–71) n=116 | 0.48 ^3^ |
| CD4+ T cell count (cells/µl) at AIDS, median (range) ^4^ | 190 (10–540) n=19 | 110 (10–720) n=91 | 0.29 ^1^ | 90 (10–850) n=19 | 97 (7–1,380) n=75 | 1.00 ^1^ |

N.A., not applicable

^1^ Mann Whitney test

^2^ Time to develop HAD after AIDS diagnosis among the cases was compared to the time from AIDS diagnosis to death or to start cART in the control group

^3^ unpaired t test

^4^ CD4+ T cell counts within 6 months to the date of AIDS diagnosis
